# To Be or Not To Be Humorous? Cross Cultural Perspectives on Humor

**DOI:** 10.3389/fpsyg.2016.01495

**Published:** 2016-10-04

**Authors:** Xiaodong Yue, Feng Jiang, Su Lu, Neelam Hiranandani

**Affiliations:** ^1^Department of Social Science, City University of Hong KongHong Kong, Hong Kong; ^2^Department of Organization and Human Resources Management, Central University of Finance and EconomicsBeijing, China; ^3^Department of Human Resources Management, University of International Business and EconomicsBeijing, China

**Keywords:** Chinese, humor perception, humor evaluation, cultural priming, Western

## Abstract

Humor seems to manifest differently in Western and Eastern cultures, although little is known about how culture shapes humor perceptions. The authors suggest that Westerners regard humor as a common and positive disposition; the Chinese regard humor as a special disposition particular to humorists, with controversial aspects. In Study 1, Hong Kong participants primed with Western culture evaluate humor more positively than they do when primed with Chinese culture. In Study 2a, Canadians evaluate humor as being more important in comparison with Chinese participants. In Study 2b, Canadians expect ordinary people to possess humor, while Chinese expect specialized comedians to be humorous. The implications and limitations are discussed.

## Introduction

On December 14, 2008, an Iraqi journalist startled attendees at a press conference at the prime minister’s palace in Baghdad, Iraq, by throwing a shoe at U.S. President George W. Bush. After the incident, Bush joked: “If you want the facts, it’s a size 10” (BBC, 2008). A few weeks later, on February 2, 2009, a student threw a shoe at Chinese Premier Wen Jiabao as he was giving a speech at the University of Cambridge. The student was removed from the lecture hall, but Premier Wen was not amused: “this despicable behavior will do nothing to hold back the friendship of the Chinese and British people” (China View, 2009). Two leaders, Western and Chinese, and two vastly different reactions to an unexpected insult, one humorous and one serious: the incidents highlight culturally different attitudes toward humor, the subject of this article.

Humor is a broad and multifaceted concept. The Oxford English dictionary defines humor as “the faculty of observing what is ludicrous or amusing or of expressing it; jocose imagination or treatment of a subject” (SOED, third edition). Humor encompasses amusement and comic reactions (Simpson and Weiner, 1989), psychological cognitive appraisals comprising perceptions of playful incongruity, mirthful emotions, and vocal-behavioral expressions of laughter (Martin, 2007, p. 10). Although humor is a universal human experience, people of different societies perceive and use humor differently (Martin, 2007; Yue, 2010). In the context of cross-cultural differences between Westerners and the Chinese, Judge Wu said: “Whereas Westerners are seriously humorous, Chinese people are humorously serious” (quoted in Kao, 1974, p. xviii).

Styles of humor are categorized as self-enhancing, affiliative, self-defeating, and aggressive (Kuiper et al., 2004; Martin, 2007). The four humor types have been investigated across cultures to show that both Westerners and Easterners are saddened and repelled by aggressive humor (Kuiper et al., 2010). North Americans react positively to self-enhancing humor, while Easterners do not (Kuiper et al., 2004; Chen and Martin, 2005). The cultural differences are attributed to the Western individualistic versus Eastern collectivistic cultural distinctions. In other words, Easterners have a collectivistic orientation that blurs the distinction between self and others so that they have weaker perceptions regarding self-oriented (self-enhancing) and other-oriented (affiliative) humor.

In general, Western individuals tolerate and use humor more than Chinese individuals do (e.g., Liao, 1998; Chen and Martin, 2007; Davis, 2011; Yue, 2011). Research has focused on specific humor styles but not on general perceptions of humor. The shoe-throwing incidents that sparked such diverse reactions inspired us to examine how people from different cultural backgrounds view humor in general, rather than focusing on the specific styles. We propose that Westerners will see humor as a positive disposition that enhances self-actualization and interpersonal relationships, and that everyone possesses the popular trait (e.g., Maslow, 1968; Martin, 2007). In contrast, the Chinese will view humor as a controversial disposition in social interactions and a personality trait possessed largely by specialists in humor-related fields (e.g., Lin, 1974; Yue, 2010, 2011; Davis, 2011; Xu, 2011). Next we present a detailed description of the two views on humor.

### The Western View on Humor

Westerners tend to take humor as a natural feature of life and to use it wherever and whenever possible (Apte, 1985). In fact, Westerners have valued humor since the era of Plato and Aristotle as a natural expression of amusement, fun, and delight in social interactions (Grant, 1924/1970). The 19th and early 20th centuries are thought to be the beginning of a golden age of humor, particularly for American society (Bier, 1968; Blair and Hill, 1978):

Humor is ubiquitous in American society and nothing escapes from becoming its target. Humor in its numerous techniques and forms is directed at the population through all conceivable channels – newsprint, magazines, books, visual and plastic arts, comedy performances, and amateur joke-telling contests, as well as many types of artifacts such as T-shirts, watches, bumper stickers, greeting cards, sculptures, toys, and so forth (Apte, 1985, p. 30).

Freud (1928) posited that humor is an effective defense mechanism against negative emotions. On one hand, laughter releases excess nervous energy; on the other hand, humor provides alternative perspectives about fear, sadness, or anger in the face of incongruous or amusing components (Martin, 2007). Early 20th century Western psychologists argued that humor and laughter enhance human health (e.g., Sully, 1902; McDougall, 1922), promote creativity (e.g., Guilford, 1950; Richards, 1990), and strengthen coping and optimism (e.g., Walsh, 1928).

Western research shows that humor could be an indispensable “panacea” in daily life to facilitate coping (e.g., Lefcourt et al., 1995; Kuiper and Martin, 1998; Moran and Massam, 1999; Lefcourt, 2001), promote impression management (e.g., Mettee et al., 1971), and enhance interpersonal attraction (e.g., Fraley and Aron, 2004). In addition, Westerners tend to regard humor as a core trait of self-actualization (Maslow, 1968; Mintz, 1983; Mindess et al., 1985) and an essential characteristic of creativity (Guilford, 1950; Sternberg, 1985).

Moreover, in the West, individuals who engage in humorous behavior are often perceived as positive and attractive (Bressler et al., 2006). Westerners tend to rate humor as an ideal and critical personal characteristic for dating or romantic partners (Hansen and Hicks, 1980; Regan and Joshi, 2003). Beyond romantic affiliations, Westerners have positive perceptions about humorous individuals. For example, a study in organizational contexts revealed that subordinates view humorous supervisors as more motivating, confident, friendly, intelligent, and pleasant leaders (Decker, 1987; Priest and Swain, 2002). Similarly, in competitive sports contexts, players wanted to play for a humorous coach and perceived the coach as competent (Grisaffe et al., 2003). In short, in Western society, people who have a sense of humor are positively perceived as more extroverted and socially desirable; in contrast, those who lack a sense of humor draw negative perceptions (Allport, 1961; Cann and Calhoun, 2001; Priest and Swain, 2002).

As such, it is no surprise that President Bush joked about the size of the shoe that was thrown at him. True to Western perceptions of humor, he demonstrated wit and charisma in the face of an embarrassing situation.

### The Chinese View on Humor

In China, humor was first documented about 2,000 years ago (Yue, 2010; Chey, 2011; Davis, 2011). The Chinese term *huaji* is regarded as an alternative word for humor meaning wit, irony, and sarcasm (Chen, 1985; Liao, 2003). The earliest form of Chinese humor could be *pai shuo*, which means small talk or jokes (see Yue, 2010, for a review). In the 1920s, Lin Yu-tang (1895–1976), a well-known writer and scholar, used the Chinese character *youmo* as the Chinese version of humor. Since then, *youmo* has widely represented wit, irony, and hilarity (Lin, 1974).

Although humor has a long past, for the past 2000 years it has been devalued under Confucianism (Lin, 1974; Yue, 2010, 2011; Xu, 2011). Lin (1974) used the term *Confucian Puritanism* to depict how humor was despised:

Confucian decorum put a damper on light, humorous writing, as well as on all imaginative literature, except poetry. Drama and the novel were despised as unworthy of a respectable scholar’s occupation...... This puritanical, austere public attitude has persisted to this day (Lin, 1974, p. xxxi).

As such, the Confucian way of a gentleman requires restraint from laughter to demonstrate dignity and social formality (Yue, 2010; Xu, 2011). The Confucian doctrine of moderation advocates against hilarious laughter because it expresses extreme emotion (Liao, 1998). The Confucian orthodox literary writings forbade humorous expressions as being beneath proper literature (Lin, 1974; Yue, 2010; Qian, 2011). Confucius even said “a man has to be serious to be respected” (Liao, 2007). As a result, the Chinese feel that they should laugh only at certain times, in conjunction with certain subjects, and only with certain people (Yue, 2011).

If they chose to laugh, Chinese people were advised to laugh gently. Chinese women were advised to cover their mouths with their hands (Lin, 1934). In short, owing to Confucian concerns for maintaining proper social order and hierarchy, proper humor is “a form of private, moderate, good-natured, tasteful, and didactically useful mirth” (Xu, 2011, p. 70). Consequently, Chinese people have long scorned public humor. Confucian moralists feared that once humorous writing styles spread, life would lose its seriousness, and sophistry would overturn orthodoxy (Yue, 2010, 2011; Sample, 2011).

Though humor has thrived in China since the downfall of the Qing dynasty (1644–1911), Chinese people are still heavily influenced by cultural biases against public humor that are deeply rooted in Confucianism (Davis, 2011; Xu, 2011). For example, humor has been consistently omitted from the list of qualities required for being a typical and creative Chinese thinker (Rudowicz and Yue, 2000, 2003; Rudowicz, 2003; Yue et al., 2006; Yue, 2011). Loud laughter tends to make Chinese people feel nervous and uncomfortable (Liao, 1998). In addition, Chinese students tend to consider themselves as being less humorous than Canadian students, and they tend to use less humor to cope with stress (Chen and Martin, 2005). Similarly, American students rated sexual and aggressive jokes as funnier than Singaporean Chinese students who preferred harmless humor (Nevo et al., 2001). Those findings support the claim that Chinese prefer a “thoughtful smile” to “hilarious laughter” (Lin, 1974). Thus, it is no surprise that Premier Wen would respond sternly to the shoe-throwing incident to keep his dignity.

Consistent with those observations, Yue (2011) systematically reviewed Chinese perceptions and identified three Chinese ambivalences toward humor. First, the Chinese tend to value humor but devalue humor as a trait of self. Chinese traditional social norms value seriousness, so Chinese people tend to fear that being humorous will jeopardize their social status. For instance, although Chinese undergraduates self-reported that humor is important in everyday life, they reported that they were not humorous themselves (Yue et al., 2006; Yue, 2011). Second, as Yue (2011) explained, being humorous is inappropriate for orthodox Chinese because Confucianism has equated humor with intellectual shallowness and social informality (Yue, 2010). For example, Chinese students do not rank humor as characteristic of an ideal Chinese personality (Rudowicz and Yue, 2003; Yue et al., 2006). Chen (1985) argued that Chinese jokes have always focused on “denial humor” that criticizes reality and “complimentary humor” that praises reality, in contrast with the “pure humor” that makes people laugh in Western jokes. Third, the Chinese tend to believe that humor is important but only for professional entertainers with exclusive expertise and special talent.

Although the four styles of humor have been examined cross-culturally, few empirical studies have examined cross-cultural differences on general humor perceptions (e.g., Nevo et al., 2001; Jiang et al., 2011). Jiang et al. (2011) found that Chinese undergraduates tended to associate humor with unpleasant adjectives and seriousness with pleasant adjectives; the opposite was true for American undergraduates. Such a finding indicates that Westerners and Chinese may hold different views toward humor in general. In addition, little work has been done to provide a comprehensive picture of the cultural differences on humor perception. Therefore, we conducted two studies to systematically verify the proposed dichotomy between the Western and Chinese view on humor.

## Overview of the Research

Two studies were conducted to examine Western versus Chinese views on humor. In Study 1, Hong Kong Chinese participants (bicultural samples) were first primed with either Western culture icons or Chinese culture icons. Then they were asked to use adjectives from a list to describe a humorous person. We expected the priming with Western culture icons would cause Hong Kong participants to assign significantly more positive adjectives, while the priming with Chinese culture icons would have the opposite effect. In Study 2a, participants from Canada and China were asked to rate the importance of humor, self-humor, and sense of humor. We expected that the Chinese would give significantly lower ratings to all three. In Study 2b, participants from Canada and China were asked to identify the names and occupations of up to three humorous persons. We expected that Canadian participants would nominate significantly more ordinary people than Chinese participants, and Chinese participants would nominate significantly more humor-relevant specialists such as comedians and cartoonists. Taken together, we hoped to find consistent findings for the proposed dichotomy between Western and Chinese views on humor.

## Study 1

We conducted Study 1 as a between subject design by priming Chinese and Western cultural differences. Bicultural Hong Kong people are considered appropriate for cultural priming studies. (For details, see Hong et al., 2000). Our purpose was to determine whether study participants exposed to pictures associated with Chinese or Western culture would be induced to perceive different qualities in a humorous person.

### Method

#### Participants and Design

Ninety-six Hong Kong college students (31 men, 65 women) were recruited. They averaged 24.01 years old (*SD* = 3.78 years). Participants were randomly assigned to two experimental groups: the Chinese picture-priming condition or the Western picture-priming condition. Following the priming (about 15 s), participants were asked to judge a humorous person by choosing from a list of 40 adjectives (Zhang et al., 1998). Oral instructions were given in Chinese and English and were counterbalanced across the priming condition to reduce potential language biases (e.g., Meier and Cheng, 2004). After the experiment, all participants were debriefed, thanked, and dismissed.

#### Materials and Procedures

##### Priming

**FIGURE 1:**
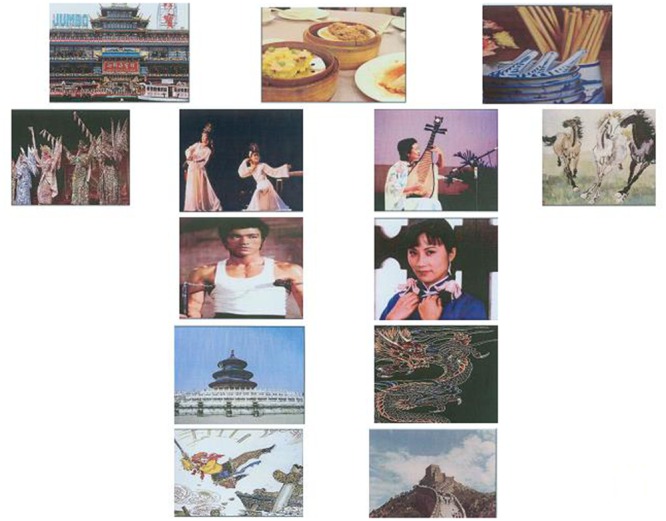
**Chinese primes**.

**FIGURE 2:**
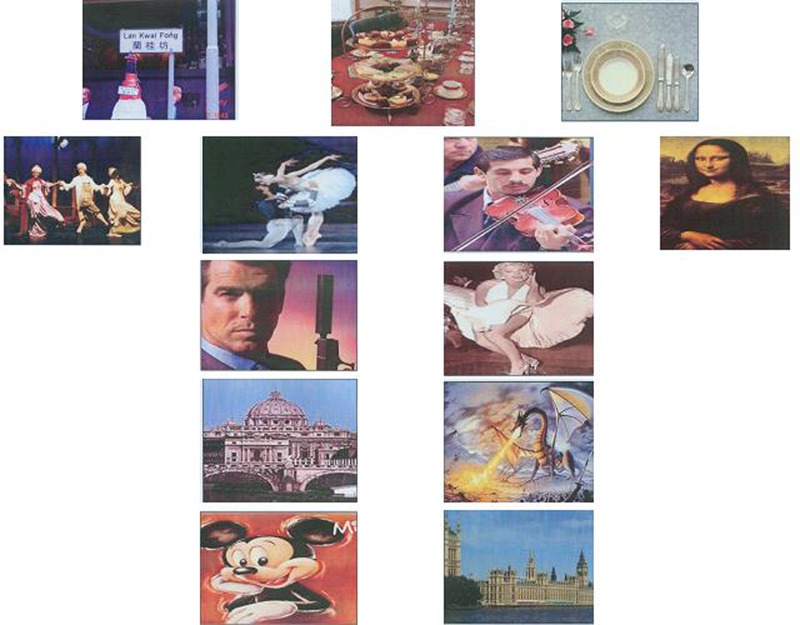
**Western primes**.

We used 26 priming pictures, 13 for each culture (**Figures 1** and **2**), from priming materials developed by Ng and Lai (2009) and based on the work of Hong et al. (2000). Moreover, the pictures were made more suitable for Hong Kong participants by adding special icons of Hong Kong culture (e.g., Dim Sum). The pictures depict culturally relevant representations of (a) food and drink, (b) music and art, (c) popular movie icons, (d) religion and legend icons; (e) and folklore and famous buildings (Ng and Lai, 2009). In accordance with common practice (Ng and Lai, 2009), the priming stimuli were presented one at a time for 5 s at a computer monitor. Participants were randomly assigned to the Western culture condition (48: 13 men, 35 women), and the Chinese culture condition (48: 18 men, 30 women). After viewing the 13 pictures, participants answered the question: “Which culture, Western or Chinese, do the pictures depict?” Then they were asked to list some of the features and to explain how the features personally influenced them.

##### Rating

The word lists were selected from Zhang et al. (1998). The adjective list was drawn up about 20 years ago when all the words were commonly used in Mainland China. We did a pilot study on 15 university students and discarded the words that were judged unfamiliar. Finally, 40 words were selected, with 20 for each positive and negative word list. Participants were asked to rate “to what degree can the word be used to depict a humorous person” on 5-point scale, from 1 = *not at all* to 5 = *very much*.

#### Results

##### Manipulation check

The check for priming showed that participants answered the questions correctly and their writings were related to each culture.

##### Rating

The scores of negative words were reverse-coded first. Then an index of humor perception was created by averaging the scores of the 40-word list. Higher scores indicated a more positive evaluation of humor. As expected, participants who were primed with Western icons showed significantly more positive evaluation of humor (*M* = 3.70, *SD* = 0.33) than those (*M* = 3.58, *SD* = 0.26) primed with the Chinese icons [*t*_(94)_ = 2.04, *p* < 0.05], *d* = 0.40.

### Discussion

Study 1 confirms the notion that the West and Chinese cultures may exert different views regarding humor (Yue, 2010, 2011). Specifically, bicultural Hong Kong participants who were primed with Chinese icons, tended to adopt a Chinese view toward humor, and consequently evaluated the humorous person less positively. On the contrary, bicultural Hong Kong participants who were primed with Western icons, were more likely to embrace a Western view toward humor and evaluate the humorous person more positively. Although the findings delighted us, we still wondered whether we could explicitly obtain such implicit Westerner versus Chinese responses toward humor. That is, would Westerners and Chinese give explicit self-reports regarding their humor perceptions? Thus, Studies 2a and 2b were conducted.

## Study 2

### Study 2a

In Study 2a, we sought to compare humor perceptions between Canadians and Chinese^1^, expecting to see more humor appreciation from Canadian participants.

### Method

#### Participants

For Study 2a, 121 Canadian undergraduates (61 women, 60 men), average 19.84 years old (*SD* = 2.54), and 121 Chinese undergraduates (48 women, 73 men), average 21.16 years old (*SD* = 1.55) were recruited.

#### Measures

Participants were given a set of questionnaires to measure their perceptions regarding humor and self-humor. One item was used to test general perceptions about humor (Yue, 2011): “How important is humor to you?” To measure the level of self-humor, we asked, “How do you rate your level of humor?” Participants responded on a ten-point scale, from 1 = l*owest* to 10 = *highest*.

The Sense of Humor Questionnaire (Herzog and Strevey, 2008) was also used to measure perceptions of humor. It includes four dimensions: humor production (e.g., “I initiate or start humorous interactions more than others do”); coping humor (e.g., “Humor helps me cope”); humor appreciation (e.g., “I appreciate those who generate humor”); and humor tolerance (e.g., “No humor topic is off-limits”). The internal reliability Cronbach’s alpha for the four dimensions was 0.87, 0.87, 0.84, 0.67, respectively.

##### Control variable

Individualism–collectivism cultural distinctions have been shown to influence perceptions of humor (e.g., Kuiper et al., 2004). In the current study, as a control variable, horizontal and vertical individualism/collectivism were assessed with the scale developed by Triandis and Gelfand (1998). The internal reliability Cronbach’s alpha for horizontal individualism, vertical individualism, horizontal collectivism, and vertical collectivism were 0.77, 0.72, 0.71, 0.69, respectively.

#### Results

All humor ratings were investigated as dependent variables, and multivariate analysis of covariance (MANCOVA) was used between the samples of Canadians and Chinese, with individualism/collectivism as a covariate variable^2^. Results showed that Canadian participants, compared with Chinese participants, evaluated humor as being significantly more important (*M*_Canadian_ = 8.56, *SD* = 1.17 vs. *M*_Chinese_ = 7.60, *SD* = 1.55, *d* = 0.70) and considered themselves as being significantly more humorous (*M*_Canadian_ = 7.28, *SD* = 1.39 vs. *M*_Chinese_ = 6.12, *SD* = 1.85, *d =* 0.71). Canadian participants were significantly higher than Chinese students on humor production (*M*_Canadian_ = 49.52, *SD* = 8.99 vs. *M*_Chinese_ = 44.85, *SD* = 8.02, *d* = 0.55), humor appreciation (*M*_Canadian_ = 55.01, *SD* = 6.30 vs. *M*_Chinese_ = 45.89, *SD* = 6.84, *d* = 0.1.39), and humor coping (*M*_Canadian_ = 45.70, *SD* = 9.82 vs. *M*_Chinese_ = 41.81, *SD* = 5.98, *d* = 0.48), but not on humor tolerance (*M*_Canadian_ = 16.43, *SD* = 5.66 vs. *M*_Chinese_ = 13.91, *SD* = 3.09, *d* = 0.55). **Table 1** displays the results in detail. The findings confirmed the previous findings and supported our hypotheses that Westerners view humor differently from Chinese (Liao et al., 2006; Chen and Martin, 2007).

**Table 1:** Cultural differences on perception of humor.

	Canadians		Chinese		
	Mean	*SD*	Mean	*SD*	*F*
Importance of humor	8.56	1.17	7.60	1.55	20.99^∗∗∗^
Rating of Self-humor	7.28	1.39	6.12	1.85	17.95^∗∗∗^
Humor production	49.52	8.99	44.85	8.02	8.80^∗∗^
Humor appreciation	55.01	6.30	45.89	6.84	75.78^∗∗∗^
Coping humor	45.70	9.82	41.81	5.98	5.13^∗^
Humor tolerances	16.43	5.66	13.91	3.09	2.66

*^∗^<0.05; ^∗∗^<0.01; ^∗∗∗^<0.001.*

### Discussion

The findings reported here confirm that Canadian and Chinese students have the expected cultural differences; that is, Canadian students rate humor as being significantly more important than Chinese students do. In addition, Canadian students consider themselves to be significantly more humorous than Chinese students do. This finding is consistent with Study 1 and further suggests that Canadians and Chinese have explicitly different views toward humor.

### Study 2b

In Study 2b, in an attempt to verify the cultural differences in perceptions about humorous people, we asked participants to give the names and occupations of up to three humorists they knew of. Aligned with our cultural suppositions, we expected that Canadians would name humorous friends and family from their everyday lives, regardless of race or occupation, whereas the Chinese would name professional humorists from the entertainment industries (Yue, 2011).

### Method

#### Participants

Studies 2a and 2b used the same participants.

#### Measures

Participants were asked to nominate up to three people they perceived as being the most humorous, to indicate whether their nominees were relatives or friends, and to identify the occupations of the humorists.

#### Results

Two research assistants helped to identify nominees’ occupations to ensure that participants answered the question correctly. The inter rater reliability was 0.97. As expected, Canadian participants nominated significantly more relatives and friends (47.09%) than did Chinese participants (14.42%). In addition, Canadian nominees had much broader occupations, such as journalists, teachers, and athletes, while the Chinese participants nominated mostly professional comedians, actors/actresses, and singers (81.73%) and rarely relatives and friends (14.42%).

**FIGURE 3:**
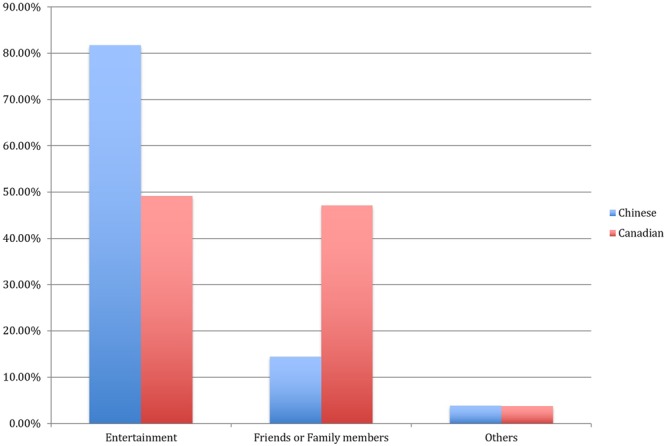
**The percentages of nominated humorists by Chinese and Canadian students**.

The percentages for occupations other than entertainment were about 1%, so we categorized those as “Others.” **Figure 3** shows distribution details.

Chi-square test results indicated a significant interaction effect between culture and category χ*^2^* = 62.21, *df* = 2, *p* < 0.001. The findings confirmed the expected cultural differences in perceptions about humor.

### Discussion

Study 2b used the nomination method and provided convergent support to the supposition that Westerners and Chinese have diverging views about humor. That is, Westerners believe that humor is a common trait among ordinary people, while the Chinese consider humor to be an extraordinary trait restricted mainly to entertainers such as comedians. As expected, the study showed that Canadians tended to see their friends and family members as examples of humorous people, while the Chinese attributed humor to professional comedians.

## General Discussion

In his book *The Psychology of Humor: An Integrative Approach*, Martin (2007) remarked: “Although humor and laughter are universal in humans and are likely a product of natural selection, the way people use and express them in a given time and place is strongly influenced by cultural norms, beliefs, attitudes, and values (p. 26).” A substantial drawback of humor research, however, is that scant attention has been paid to cultural influences on humor perception, particularly differences between Western and Chinese culture, largely because individuals absorb cultural attitudes so that their views are colored by their exposure to their Western or Eastern culture (Martin, 2007). Thus, we can reasonably expect that the current positive view of humor reflects cultural bias rooted in the Western world (Martin, 2007).

In this paper, we offer a distinction between Western and Chinese views of humor. Most current investigations tend to take the Western view that sees humor as positive and beneficial (e.g., Galloway and Cropley, 1999; Martin, 2002). Our findings show that bicultural Hong Kong study participants primed with Chinese culture attributed less positive words to humorists, and more positive words when primed with Western culture. Moreover, compared with Canadians, Chinese attributed significantly less value to humor and rated themselves as being much less humorous. They also nominated significantly more professional humorists rather than friends and family as representatives of humor. These findings provide converging evidence that Chinese and Westerners tend to view humor quite differently for both themselves and others. The findings echo and explain previous findings that Chinese hold negative implicit attitudes toward humor (Jiang et al., 2011), deny humor as an ideal component or an indication of creativity (Rudowicz and Yue, 2000, 2003; Yue, 2011), and use less humor than Westerners do (Chen and Martin, 2005, 2007).

### Implications

First, this study provides a conceptual model of cross-cultural research on humor. Only a few studies have considered Western and Chinese cultural differences regarding the use and toleration of humor (e.g., Chen and Martin, 2005, 2007; Jiang et al., 2011). The present study offers an appropriate broader perspective for explaining cultural influences on humor perception and integrating previous humor studies.

Second, the study augments understandings regarding the cross-cultural meanings and implications of humorous messages. Although previous research showed that Westerners are more prone than Easterners to use self-enhancing humor, the current study further suggests that Easterners generally deprecate humor while Westerners value it (Kuiper et al., 2010). The different cultural views may lead to cultural biases. For instance, Chinese children tend to see humor as aggressive and disruptive (Chen et al., 1992). Consequently, Americans and Chinese who try to communicate cross culturally many find that cultural variations regarding humor may disrupt their communications.

Third, we are not saying that Chinese people lack humor. On the contrary, abundant evidence shows that humor has been common and popular throughout Chinese history (Xiao, 1996). Instead, we argue that Confucian biases have caused public humor to be more “in deeds than in words, more practiced than preached” in China (Kao, 1974, p. xxii). Thus, before a Chinese leader such as Wen Jiabao could joke about an embarrassing situation, the general Chinese population must first see humor as positive and desirable. They must go beyond Confucian puritanism that frowns on humor and instead learn to value, appreciate, and use humor whenever and wherever possible (Chen and Martin, 2005; Yue, 2010, 2011).

As Lin Yutang said, “the secret of humor is to be natural and to be oneself, to face oneself in the mirror and to tear down the hypocritical disguise” (Qian, 2011, p. 211). After all, the ability to laugh at ourselves comes from broad-minded detachment regarding our own imperfections. And this remains to be further examined in later studies.

### Limitations and Future Directions

The current study has several inherent limitations that should be noted. First, Hong Kong Chinese, not Mainland Chinese, participated in Study 2. As Hong Kong is highly westernized, the students may not perfectly represent Chinese society. The findings may lend credence to the expectation that Mainland Chinese will show even greater differences with Westerners. Consequently, future investigations should replicate the current findings with more Mainland Chinese samples. Second, although the results of Study 2a are consistent with what we found in Studies 1 and 2b, it still bears the contamination of culture-related response biases (e.g., Chen et al., 1995; Heine et al., 2002). As we know, people from different cultures tend to use different referents in their self-reported values. Thus, Canadians in the current research evaluated humor in comparison with other Canadians, whereas Chinese evaluated humor in comparison with other Chinese. In addition, Chinese are more likely than Canadians to use the midpoint on self-reported scales (e.g., Markus and Kitayama, 1991; Chen et al., 1995). For future investigations, it would be necessary to measure participants’ evaluation on both humor and seriousness. In doing so, we can examine the differences of rating patterns instead of direct rating scores between Chinese and Canadians. In other words, it allows us to investigate whether Canadian participants would rate humor as being more important to them than being serious, while the opposite pattern would be true for Chinese participants. Third, the nomination method (Study 2b) helped to validate the two contrasting views of humor between the West and the East, but social media influences and entertainment development could be confounding factors (e.g., Buijzen and Valkenburg, 2004). Therefore, future studies should control for interfering factors. Fourth, all samples were confined to university students. For broader generalization, future studies should recruit participants of various ages and from various backgrounds.

## Conclusion

The current research provides new evidence and a broader perspective for studying cultural differences regarding humor perception. Westerners view humor as a commonly owned trait and as a positive disposition for self-actualization. In contrast, the Chinese consider humor to be restricted to humor professionals and less desirable for social interactions. Two studies employing priming paradigm, questionnaire measurement, and nomination technique presented in this paper reveal the dichotomy. We hope that these findings stimulate future studies that venture further into the frontier area of humor.

## Author Contributions

All authors conceptualized the manuscript, XY and FJ wrote the first complete draft, XY and SL contributed additional writing, FJ, SL, and NH contributed data collection and analysis, all authors edited the manuscript and approved the final version.

## Conflict of Interest Statement

The authors declare that the research was conducted in the absence of any commercial or financial relationships that could be construed as a potential conflict of interest.

## Footnotes

1 Please be noted that all Chinese participants are Hong Kong Chinese. The potential impacts are discussed in discussion part.

2 The results of individualism-collectivism ratings: horizontal individualism, *M*_Canadian_ = 30.92, *SD* = 3.95 vs. *M*_Chinese_ = 29, *SD* = 3.50, *t* = 4.01, *p* < 0.0001, *d* = 0.51; vertical individualism, *M*_Canadian_ = 25.77, *SD* = 5.85 vs. *M*_Chinese_ = 24.96, *SD* = 4.11, *t* = 1.25, *p* = 0.21, *d* = 0.16; horizontal collectivism, *M*_Canadian_ = 30.15, *SD* = 4.67 vs. *M*_Chinese_ = 29.64, *SD* = 3.51, *t* = 0.96, *p* < 0.34, *d* = 0.12; vertical collectivism, *M*_Canadian_ = 23.35, *SD* = 4.56 vs. *M*_Chinese_ = 27.66, *SD* = 3.42, *t* = -8.30, *p* < 0.001, *d* = -1.07.
